# si-SNHG5-FOXF2 inhibits TGF-β1-induced fibrosis in human primary endometrial stromal cells by the Wnt/β-catenin signalling pathway

**DOI:** 10.1186/s13287-020-01990-3

**Published:** 2020-11-11

**Authors:** Limin Liu, Guobin Chen, Taoliang Chen, Wenjuan Shi, Haiyan Hu, Kaijing Song, Ruichun Huang, Huihua Cai, Yuanli He

**Affiliations:** 1 Department of Obstetrics and Gynecology, Zhujiang Hospital, Southern Medical University, Guangzhou, China; 2 Department of Obstetrics and Gynecology, Affiliated Shenzhen Maternity & Child Healthcare Hospital, Southern Medical University, Shenzhen, China; 3 The National Key Clinical Specialty, The Engineering Technology Research Center of Education Ministry of China, Guangdong Provincial Key Laboratory on Brain Function Repair and Regeneration, Department of Neurosurgery, Zhujiang Hospital, Southern Medical University, Guangzhou, China; 4 Department of Obstetrics and Gynecology, Guangdong Provincial People’s Hospital, Guangdong Academy of Medical Sciences, Guangzhou, China

**Keywords:** IUA, TGF-β1, FOXF2, SNHG5, ECM, Fibrosis

## Abstract

**Background:**

Intrauterine adhesions (IUAs) are manifestations of endometrial fibrosis characterized by inflammation and fibrinogen aggregation in the extracellular matrix (ECM). The available therapeutic interventions for IUA are insufficiently effective in the clinical setting for postoperative adhesion recurrence and infertility problems. In this study, we investigated whether si-SNHG5-FOXF2 can serve as a molecular mechanism for the inhibition of IUA fibrosis ex vivo.

**Methods:**

FOXF2, TGF-β1 and collagen expression levels were measured by microarray sequencing analysis in three normal endometrium groups and six IUA patients. We induced primary human endometrial stromal cells (HESCs) into myofibroblasts (MFs) to develop an IUA cell model with various concentrations of TGF-β1 at various times. Downstream target genes of FOXF2 were screened by chromatin immunoprecipitation combined with whole-genome high-throughput sequencing (ChIP-seq). We investigated ECM formation, cell proliferation and Wnt/β-catenin signalling pathway-related proteins in primary HESCs with FOXF2 downregulation by quantitative reverse transcription-polymerase chain reaction (qRT-PCR), western blotting (WB), immunohistochemistry (IHC), flow cytometry, ethylenediurea (EdU) and CCK8 assays. We identified long noncoding RNAs (lncRNA) SNHG5 as the upstream regulatory gene of FOXF2 through RNA immunoprecipitation (RIP), RNA pulldown and fluorescence in situ hybridization (FISH). Finally, we examined FOXF2 expression, ECM formation, cell proliferation and Wnt/β-catenin signalling pathway-related proteins in primary HESCs upon FOXF2 downregulation.

**Results:**

FOXF2 was highly expressed in the endometrium of patients with IUA. Treatment of primary HESCs with 10 ng/ml TGF-β1 for 72 h was found to be most effective for developing an IUA cell model. FOXF2 regulated multiple downstream target genes, including collagen, vimentin (VIM) and cyclin D2/DK4, by ChIP-seq and ChIP-PCR. FOXF2 downregulation inhibited TGF-β1-mediated primary HESC fibrosis, including ECM formation, cell proliferation and Wnt/β-catenin signalling pathway-related protein expression. We identified lncRNA SNHG5 as an upstream gene that directly regulates FOXF2 by RIP-seq, qRT-PCR, WB and FISH. SNHG5 downregulation suppressed FOXF2 expression in the IUA cell model, resulting in synergistic repression of the Wnt/β-catenin pathway, thereby altering TGF-β1-mediated ECM aggregation in endometrial stromal cells ex vivo.

**Conclusions:**

Regulation of the Wnt/β-catenin signalling pathway and ECM formation by si-SNHG5-FOXF2 effectively inhibited the profibrotic effect of TGF-β1 on primary HESCs. This finding can provide a molecular basis for antagonizing TGF-β1-mediated fibrosis in primary HESCs.

## Background

Intrauterine adhesions (IUAs) are partial or total occlusions of the uterine cavity caused by disordered repair after endometrial injury; they are often accompanied by complications, such as hypomenorrhoea, amenorrhoea, infertility and recurrent miscarriage [1–3]. At present, IUAs remain a worldwide challenge and seriously threaten the fertility and reproductive health of women, thereby affecting family stability and harmony in society. Although combined hysteroscopic synechialysis with a series of postoperative adjuvant strategies can normalize the morphology of the uterine cavity to various extents and can even increase or restore the menstrual flow of some patients, the available therapeutic interventions for IUA are either insufficiently effective or unsound in the clinical setting, with the main problems including postoperative adhesion recurrence and infertility [4–6]. Postoperatively, the recurrence rate of IUAs is as high as 62.5% [7], whereas the overall pregnancy rate is only 42.8–66.1% [8]. According to China’s national conditions and humanistic ethics, stem cell use in reproduction and surrogacy is not legally allowed; therefore, focusing on the biological mechanism and aiming to seek targeted treatments for endometrial fibrosis during the formation of IUA should be considered priorities.

The main pathological changes that occur in IUAs are inflammation and fibrinogen accumulation of the extracellular matrix (ECM), which in turn leads to endometrial fibrosis [2, 9]. Transforming growth factor-β1 (TGF-β1) is recognized as a central profibrotic factor that can activate multiple signalling pathways in a process known as pathway crosstalk; bring about a complex set of interactions by the mitogen-activated protein kinase (MAPK), ERK and Wnt/β-catenin signalling pathways; and thereby induce abnormal fibrinogen secretion [10, 11]. In addition, TGF-β1 can induce the transformation of various cell types, such as vascular endothelial cells, fibroblasts (FBs) and renal tubular epithelial cells, into α-smooth muscle actin (α-SMA)-expressing myofibroblasts (MFs). MFs, which exhibit characteristics of smooth muscle cells and FBs, are generally considered the key source of ECM production during tissue fibrosis [12–14]. It has been confirmed that TGF-β1 induces the epithelial-mesenchymal transition (EMT) and causes ECM to be excessively deposited as collagen [15–17]. According to recent studies, Wnt/β-catenin signalling and TGF-β1 are the most powerful mediators that promote EMT; both increase the secretion of collagen and other ECM proteins by mesenchymal cells, inhibit ECM degradation and ultimately result in ECM deposition in damaged tissues and organs [11, 18, 19]. High expression of TGF-β1 has been demonstrated both in clinical samples from humans with IUAs and in animal IUA models [20]. Using TGF-β1 to induce the transformation of primary human endometrial stromal cells (HESCs) into MFs, we established an IUA cell model and used it to explore the underlying mechanism of inhibition of TGF-β1-mediated endometrial fibrosis ex vivo. Intriguingly, microarray sequencing analysis showed that the transcription factor forkhead box F2 (FOXF2) was highly expressed in clinical IUA samples and in the IUA cell model, implicating FOXF2 in the development of IUAs.

The FOXF2 gene encodes a DNA-binding protein that has a molecular weight of approximately 46 kDa and contains 444 amino acids [21]. FOXF2 is a specific mesenchymal transcription factor that is often expressed in mesenchymal cells adjacent to the epithelium in tissues and organs, such as the respiratory tract, urinary tract and digestive tract, and has been implicated in embryo and tissue development [22, 23], ECM synthesis [24] and EMT [25, 26]. Specifically, FOXF2 maintains cell and tissue homeostasis by regulating cell polarity and plays an important role in embryonic development and tissue differentiation [27]. Studies have reported that downregulation of FOXF2 results in a deficiency of collagen synthesis [24]. Nevertheless, the role and related mechanism of FOXF2 in IUAs have not yet been clearly illustrated.

The FOXF2 genes in humans and mice are located on chromosomes 6p25.3 and 13, respectively, and the FOXF2 proteins encoded by these two genes are 94.6% homologous [28]. Studies have demonstrated that FOXF2 gene-knockout mice exhibit intestinal malformations, colon remodelling [29] and abnormal development of the genitals and palate [30, 31]. FOXF2^−/−^ mice die at birth due to a lack of exons encoding the DNA-binding domain [23]. FOXF2 is also a key protein in cochlear development, and its dysfunction leads to cochlear malformations and deafness in humans and mice [32]. Thus, given the crucial role of the FOXF2 transcription factor in embryonic development, more attention should be paid to identifying alternative therapeutic approaches to inhibit fibrosis than to developing approaches that directly target FOXF2 in vivo.

In recent years, researchers in China and abroad have given increasing attention to long noncoding RNAs (lncRNAs) associated with fibrosis. lncRNAs (a collective name for transcripts greater than 200 nucleotides in length that do not encode proteins) participate in and control biological processes by regulating gene expression at almost all levels, including the epigenetic, transcriptional and posttranscriptional levels [33]. Studies of lncRNA may yield new opportunities for the diagnosis and treatment of human diseases. In this study, we identified lncRNA SNHG5 as the upstream regulatory gene of FOXF2 through RNA immunoprecipitation (RIP) and established its complete molecular mechanism of action to serve as the basis for subsequent experiments ex vivo.

Small nucleolar RNA (snoRNA) host gene 5 (SNHG5), also known as U50HG, is 524 base pairs (bp) in length and comprises six exons and two snoRNAs, U50 and U50′ [34, 35]. Many studies have addressed the correlation between SNHG5 and cancer in various organs. On the one hand, SNHG5 was found to be expressed at low levels in gastric cancer and to suppress the ability of gastric cancer cells to migrate and proliferate both in vitro and in vivo by inhibiting the translocation of MTA2 (metastasis-associated gene2) into the nucleus through interacting with MTA [36]. On the other hand, SNHG5 was shown to have a cancer-promoting effect, as knockdown of SNHG5 inhibited the proliferation and apoptosis of breast cancer or colorectal cancer cell lines [35, 37]. At the same time, SNHG5 was confirmed to play a pro-cancer role in hepatocellular carcinoma by upregulating GSK3β, activating the Wnt/β-catenin pathway and promoting EMT [38]. Therefore, the role of SNHG5 in cancer remains controversial and is still a subject of ongoing research. The possible role of SNHG5 in IUAs has not been explored to date. In our study, SNHG5 downregulation suppressed FOXF2 expression in the IUA cell model, resulting in synergistic repression of the Wnt/β-catenin signalling pathway and thereby altering TGF-β1-mediated ECM aggregation in endometrial stromal cells ex vivo. These results indicate that si-SNHG5 downregulates FOXF2 and the Wnt/β-catenin signalling pathway and that it can provide a molecular basis for antagonizing TGF-β1-mediated fibrosis in primary HESCs.

In previous studies, using an established IUA animal model, we demonstrated that the classical fibrogenic factor TGF-β1 participates in the pathogenesis of IUAs via the TGF-β/Smad signalling pathway [39, 40]. The current study aimed to further investigate the molecular mechanism of fibrosis in the pathogenesis of IUA and to provide valuable proof for exploring new therapeutic opportunities for the inhibition of TGF-β1-induced IUA.

## Materials and methods

### Clinical samples

For the IUA group, the endometria of six patients with IUA were collected. For the normal control group, fresh endometrial tissue was collected from six patients who underwent hysterectomy because of cervical intraepithelial neoplasia or subserosal fibroids or hysteroscopic endometrial biopsy due to infertility (three of normal endometrium groups were taken in microarray sequencing analysis). The patients were 30–45 years of age and had regular menstrual cycles (25–32 days). No hormones or intrauterine devices (IUDs) were used by the patients during the 3 months prior to surgery, and postoperative pathology suggested no endometrial lesions. Endometrial extraction was reviewed and approved by the Ethics Committee of Zhujiang Hospital of Southern Medical University, and written informed consent was obtained from each patient prior to surgery.

### Immunohistochemistry (IHC)

Tissues were fixed, paraffin-embedded and sectioned (5-μm continuous sections). H&E staining and Masson staining were performed. Paraffin sections were deparaffinized and rehydrated. The sections were heated in sodium citrate buffer in a microwave for antigen retrieval, washed with phosphate-buffered saline (PBS) for 5 min (three times) at room temperature, incubated in 3% H_2_O_2_ at room temperature for 25 min, washed with PBS for 5 min (three times) and blocked and incubated in goat serum for 30 min. The primary antibody was added dropwise (anti-FOXF2 antibody, 1:50, Abcam, Ab194427), and the sections were incubated at 4 °C overnight. The next day, the primary antibody was discarded, and the sections were washed with PBS for 5 min (three times). Then, 50 min after the addition of secondary antibody (1:200, SignalStain® Boost IHC Detection Reagent, CST, USA), the sections were washed with PBS for 5 min (three times). Finally, freshly prepared diaminobenzidine (DAB) was added for colour development, and the sections were counterstained with haematoxylin (Solarbio, China), dehydrated and mounted with neutral gum. All slides were observed and photographed under a microscope (× 200 or × 400) by a blinded investigator.

### Extraction and culture of primary HESCs

Primary HESCs were extracted by 0.2% type I collagenase digestion and filtered through a sieve. Ophthalmic scissors were used to mince the endometrial tissues obtained by curettage. A total of 4–5 ml of 0.2% type I collagenase was added, and the tissue was digested in a 37 °C constant-temperature water bath for 60 min. The solution was filtered through a 200- to 400-mesh sieve, and the suspension was collected. After centrifugation of the suspension at 1000 rpm for 5 min, the supernatant was discarded, and the cells were resuspended in complete medium and placed in a 37 °C, 5% CO_2_ incubator. After 6–8 h, the medium was replaced (nonadherent cells were also removed), and the purified endometrial stromal cells were obtained. The medium was replaced every 2–3 days, and the cells were passaged at a 1:3–1:4 ratio and cryopreserved [41].

### Immunocytochemistry (ICC) and immunofluorescence (IF)

Cells were fixed in 4% paraformaldehyde for 4 min, washed with PBS three times, incubated with 0.5% Triton-100 at room temperature for 20 min and washed with PBS three times. The cells were blocked in bovine serum albumin (BSA; 5%) for 20 min at room temperature, and the blocking solution was then removed. Antibodies against vimentin and CK-18 were added; instead of a primary antibody, PBS was added to the negative control. The cells were incubated overnight in a humidified incubator at 4 °C. The next day, the primary antibody was discarded, the cells were washed three times with PBS, and a goat anti-rabbit secondary antibody was added. The cells were incubated with the secondary antibody at 37 °C for 20 min, followed by three washes with PBS. Horseradish peroxidase-conjugated avidin (HRP-avidin, SABC) was added, and the cells were incubated at 37 °C for 20 min and then washed with PBS four times. DAB staining was performed in the dark at 37 °C for 5–10 min, followed by termination of the reaction with deionized (DI) water. The nuclei were counterstained with haematoxylin and incubated at 37 °C for 2 min. Staining was terminated with DI water. The samples were dehydrated using a conventional ethanol gradient (75–85–95–100%) for 2 min at each percentage and vitrified by dimethylbenzene for 1 min. The sections were observed and photographed under an inverted microscope (× 100).

Cells were fixed in 4% paraformaldehyde for 15 min, washed with PBS for 5 min (three times), incubated in cell membrane permeabilization solution containing 0.5% Triton X-100 for 10 min, washed with 250 μl of PBS for 5 min (three times) and blocked in 5% BSA for 1 h. The blocking solution was removed, and the cells were incubated in primary antibody at 4 °C overnight. The next day, the cells were washed with PBS for 5 min (three times) and incubated in the dark for 1–2 h with secondary antibodies conjugated to Alexa FluorTM 488 or Alexa FluorTM 633 (Thermo Fisher, USA). The secondary antibodies were removed, and the cells were washed with 250 μl PBS for 5 min (three times). The cell nuclei were stained with 4′ 6-diamidino-2-phenylindole (DAPI) for 5 min, and the DAPI was removed. The cells were washed with 250 μl of PBS for 5 min (three times), placed on glass slides (cells facing down) and labelled; glycerin was then added to seal the slides. The slides were observed and photographed under an upright or inverted fluorescence microscope using laser scanning confocal microscopy (LSCM) (Carl Zeiss, LSM 880, Germany) [42] (× 630 or × 1000).

### Development of an IUA cell model (TGF-β1-treated HESCs)

TGF-β1 (0, 2.5, 5, 10, 20 and 40 ng/ml) was applied to HESCs for 72 h. The mRNA and protein expression levels of COL1A1, α-SMA, COL5A2 and FOXF2 were detected by qRT-PCR, WB and IF, and cell proliferation and apoptosis were detected by flow cytometry. ECM formation, FOXF2 expression and cell proliferation were most prominent after treatment of the cells with 10 ng/ml TGF-β1. HESCs were then treated with 10 ng/ml TGF-β1 for 0, 24, 48 and 72 h. The mRNA and protein expression levels of COL1A1, α-SMA, COL5A2 and FOXF2 were detected by qRT-PCR and WB. Treatment of HESCs with 10 ng/ml TGF-β1 for 72 h was found to be the optimal condition for development of an IUA cell model.

### qRT-PCR

RNA was extracted using TRIzol (Invitrogen, USA), and the purity and concentration of the RNA were determined using a UV spectrophotometer. RNA was reverse-transcribed into cDNA using a PrimeScriptTM RT reagent kit (Takara, Japan). Amplification was performed according to the SYBR® Premix Ex Taq (Takara, Japan) protocol using a CFX96TM Real-Time PCR Detection System (Bio-Rad). Relative quantification was performed using the 2^−ΔΔCt^ method. The 2^−ΔΔCt^ value represents the expression level of a target gene in each group relative to the expression level of the internal reference gene. ΔΔCt = (Ct target gene − Ct reference gene) experimental group − (Ct target gene − Ct reference gene) control group. The Ct value was automatically determined based on the amplification curve. All of the reactions were performed in triplicate. The primer sequences are listed in Supplementary Table 1.

### Western blotting (WB)

Total proteins were extracted from primary HESCs or endometrial tissue using RIPA lysis buffer (Beyotime Biotechnology, Shanghai, China). The BCA-100 protein quantitation method (Keygen Biotech, China) was used to determine the protein concentration. An 8–12% separation gel and a 5% stacking gel were prepared for electrophoresis. After separation, the separated proteins were transferred to a membrane by the wet transfer method. The membrane was blocked in 5% skim milk (total protein) and BSA (phosphorylated protein) for 1 h, and primary antibodies (details are shown in supplementary materials) were then added. The membrane was stored at 4 °C overnight and then washed with 1× Tris-buffered saline-Tween (TBST) for 5 min (three times). After addition of the appropriate secondary antibodies, the membrane was incubated at room temperature for 1–2 h and then washed with 1× TBST for 5 min (three times). The membrane was incubated in electrochemiluminescence (ECL) substrate (Millipore, USA), and the blots were developed in an ultrasensitive chemiluminescence imaging system (Bio-Rad) (the antibodies used in these experiments are provided in Supplementary Table 2).

### Flow cytometry

For *cell cycle analysis*, cells were trypsinized, resuspended in PBS and washed twice. The cells were incubated in precooled 70% ethanol, and the ethanol was discarded after centrifugation. The cells were then resuspended in PBS and washed twice. Propidium iodide (PI, 450 μl)/RNase (50 μl) staining buffer (BD PharmingenTM, USA) was added, and the reaction was allowed to proceed at room temperature for 30 min in the dark. The samples were filtered through a 200-mesh nylon sieve and then sent to a flow detection tube. A FACS flow cytometer was used (Verse, BD, USA).

For apoptosis detection, cells were digested in 0.25% trypsin without ethylenediaminetetraacetic acid (EDTA). The cells were resuspended in PBS and washed twice; 100 μl of 1× binding buffer, 5 μl of FITC-annexin V (eBioscience, USA) and 10 μl of PI (eBioscience, USA) were then added to each sample in the dark. The dye was mixed thoroughly in the dark at room temperature for 15 min and mixed with PBS (400 μl/sample). The final volume was 500 μl/sample. The samples were filtered through a 200-mesh nylon sieve into a flow detection tube. Each sample was labelled and detected within 1 h of loading.

### Chromatin immunoprecipitation (ChIP)

Cells were fixed with 1% formalin to crosslink the protein and DNA. Glycine (Sigma) at a final concentration of 0.125 mol/L was used to terminate the crosslinking reaction. One millilitre of precooled PBS + 1× protein inhibitor cocktail was used to wash the cells. After centrifugation, the supernatant was discarded. Sodium dodecyl sulfate (SDS) lysis buffer (1 ml per 1 × 10^7^ cells) was then added, and the cells were incubated on ice for 10 min. The chromatin was processed by ultrasonic fragmentation to obtain 200–1500-bp DNA-protein fragments. A total of 5 μl of the supernatant was taken as the input group; 45 μl of the supernatant was diluted in 450 μl of 1× IP dilution buffer, and 500 μl of diluted lysate was added to each IP sample for insertion into the plug spin column. The primary antibody (negative control IP, 1–2 μl of rabbit anti-human IgG; target-specific IP, 1–10 μg of rabbit anti-human polyclonal FOXF2 antibody) was added, and the samples were mixed thoroughly at 4 °C overnight. Then, 20 μl of ChIP-grade protein A/G plus agarose was added to the immunoprecipitation reaction, and the mixture was incubated at 4 °C in a shaker for 1 h. The cells were washed and eluted according to the instructions. The IP group was mixed thoroughly with 2 μl of RNase A and 5 μl of proteinase K. A total of 150 μl of 1× IP elution buffer, 2 μl of RNase A and 5 μl of proteinase K were added to the input group samples. Decrosslinking of the protein-DNA complex was conducted in a metal bath at 65 °C for 3 h. The DNA fragments were purified using a kit for high-throughput sequencing and qRT-PCR.

### Cell transfection

For the prevention group, HESCs were incubated overnight to allow them to reach 20–30% confluence and then cultured in serum-free medium for 24 h to synchronize the cell cycle. siRNA and si-NC designed by GenePharma (Shanghai, China) were transfected into the cells using Lipofectamine® 3000 (Invitrogen; Thermo Fisher Scientific) according to the manufacturer’s instructions. Twelve hours after transfection, the HESCs were stimulated with culture medium containing 10 ng/ml TGF-β1 for 72 h.

For the treatment group, after achieving a synchronous cell cycle, HESCs were treated with 10 ng/ml TGF-β1 for 48 h. The HESCs were then transfected with siRNA or si-NC for 12 h and maintained in medium containing 10 ng/ml TGF-β1 for 36 h.

### Ethylenediurea (EdU) and CCK8 assays

#### EdU assay

Cells were incubated for 4 h in 100 μl of complete medium (without antibody) to which 0.2 μl of EdU working solution (CWBiotech) had been added. They were then fixed in 4% paraformaldehyde for 15 min, in 50 μl of glycine for 5 min, and washed twice with 3% BSA. To permeabilize the cell membrane, the cells were incubated in 0.5% Triton X-100 for 20 min and washed twice with 3% BSA. According to the instructions supplied by the manufacturer, the prepared mixture was added for 30 min, and the cells were washed twice with 3% BSA and once with PBS. The final concentration of Hoechst 33342 was 5 μg/ml. The cells were incubated in the dark for 15 min and washed twice with PBS. Images were captured using an inverted fluorescence microscope in a darkroom (× 100).

#### CCK8 assay

Six hours after the cells were seeded into a 96-well plate, the supernatant was removed, leaving only the cells that adhered tightly to the wells. CCK8 (10 μl) and basal medium (90 μl) were added to each well to yield a total volume of 100 μl. A blank control plate containing no cells but the same volumes of CCK8 and basal medium was also prepared. The cells and control plates were incubated for 2–3 h, and the optical density (OD) of each well at a wavelength of 450 nm was measured in the dark every 30 min. A growth curve was plotted according to the measured OD values.

### RIP and RNA pulldown assays

#### RIP

The RIP experiment was performed using a RIPTM RNA-binding protein immunoprecipitation kit (Millipore, USA) according to the manufacturer’s instructions. Approximately 5 μg of antibody (target protein, FOXF2; negative control protein, rabbit IgG; positive control protein, SNRNP70) was added, and the sample was incubated with protein G magnetic beads. After the addition of cell lysis buffer, the coprecipitated RNA was pulled down with protein G beads, followed by high-throughput sequencing.

#### RNA pulldown

Biotin-labelled SNHG5 was synthesized using T7 RNA polymerase in a biotin RNA-labelled mixture (Roche, USA) and incubated with cell lysate for 4 h. After overnight incubation with streptavidin-coated magnetic beads (Thermo, USA), biotin-labelled SNHG5 protein was pulled down. Specific bands were identified by sodium dodecyl sulfate-polyacrylamide gel electrophoresis (SDS-PAGE), silver staining and WB. The primer sequences are listed in Table S1 in the supplementary materials.

### Fluorescence in situ hybridization (FISH)

Cells on slides were fixed in 4% paraformaldehyde for 10 min and washed with PBS for 5 min (three times). The cells were incubated in precooled cell membrane permeabilization solution (0.5% Triton X-100) at 4 °C for 5 min and then washed with PBS for 5 min (three times). A total of 200 μl of prehybridization solution was added, and the cells were incubated at 37 °C for 30 min. All subsequent operations were conducted in the dark. A total of 2.5 μl of 20 μM lncRNA SNHG5 FISH Probe Mix stock solution was added to 100 μl of hybridization solution, and the cells were incubated with the solution in a 37 °C incubator overnight. The slides were then washed with hybridization solution I for 5 min at 42 °C (three times), once with hybridization solution II at 42 °C, once with hybridization solution III and once with PBS. After DAPI staining for 10 min, the slides were washed with 500 μl of PBS for 5 min (three times). The slides were removed, mounted onto glass in enhanced fluorescence signal substrate and photographed under an inverted LSCM (630×).

### Separation of the nucleus and cytoplasm

The cells were washed twice with precooled PBS, resuspended in cell fractionation buffer and incubated on ice for 5–10 min or until a clear solution was obtained. The cells were centrifuged at 500×*g* for 5 min at 4 °C to precipitate the nuclei; the cytoplasm remained in the top fraction. An equal volume of 2× Lysis/Binding Solution was added to the cytoplasm. An equal volume of anhydrous ethanol was added. The sample was filtered, and the filtrate was discarded. The sample was washed once with Wash Solution 1 and twice with Wash Solution 2/3. Elution solution that had been heated to 95 °C was then added. The sample was centrifuged for 30 s to obtain the RNA. Elution solution was added again, and the sample was centrifuged for 30 s. The nuclear RNA and cytoplasmic RNA were stored at − 80 °C or used for qRT-PCR.

### Protein-protein interaction (PPI) network construction

The PPI information was predicted using the Search Tool for the Retrieval of Interacting Genes (STRING) online database (http://string-db.org). Analysing the functional interactions among proteins could offer insights into the mechanisms of occurrence or development of IUA. To evaluate the potential PPI interaction, a PPI network of differentially expressed genes (DEGs) was mapped using the STRING database, and relationships with a combined score > 0.4 were considered statistically significant.

### Statistical analysis

Statistical analysis was performed using SPSS 20.0 (Chicago, USA) statistical software. One-way analysis of variance (ANOVA) was used to evaluate differences between two or multiple groups. Measurement data are expressed as the means ± standard error of the mean (SEM) obtained in one representative experiment out of three independent experiments. *P* < 0.05 was considered statistically significant. Figures were generated with GraphPad Prism 7 (GraphPad Software, USA) and Adobe Illustrator CS6 (Adobe, USA).

## Results

### FOXF2 is highly expressed in the endometrium of patients with IUA

**Fig. 1:**
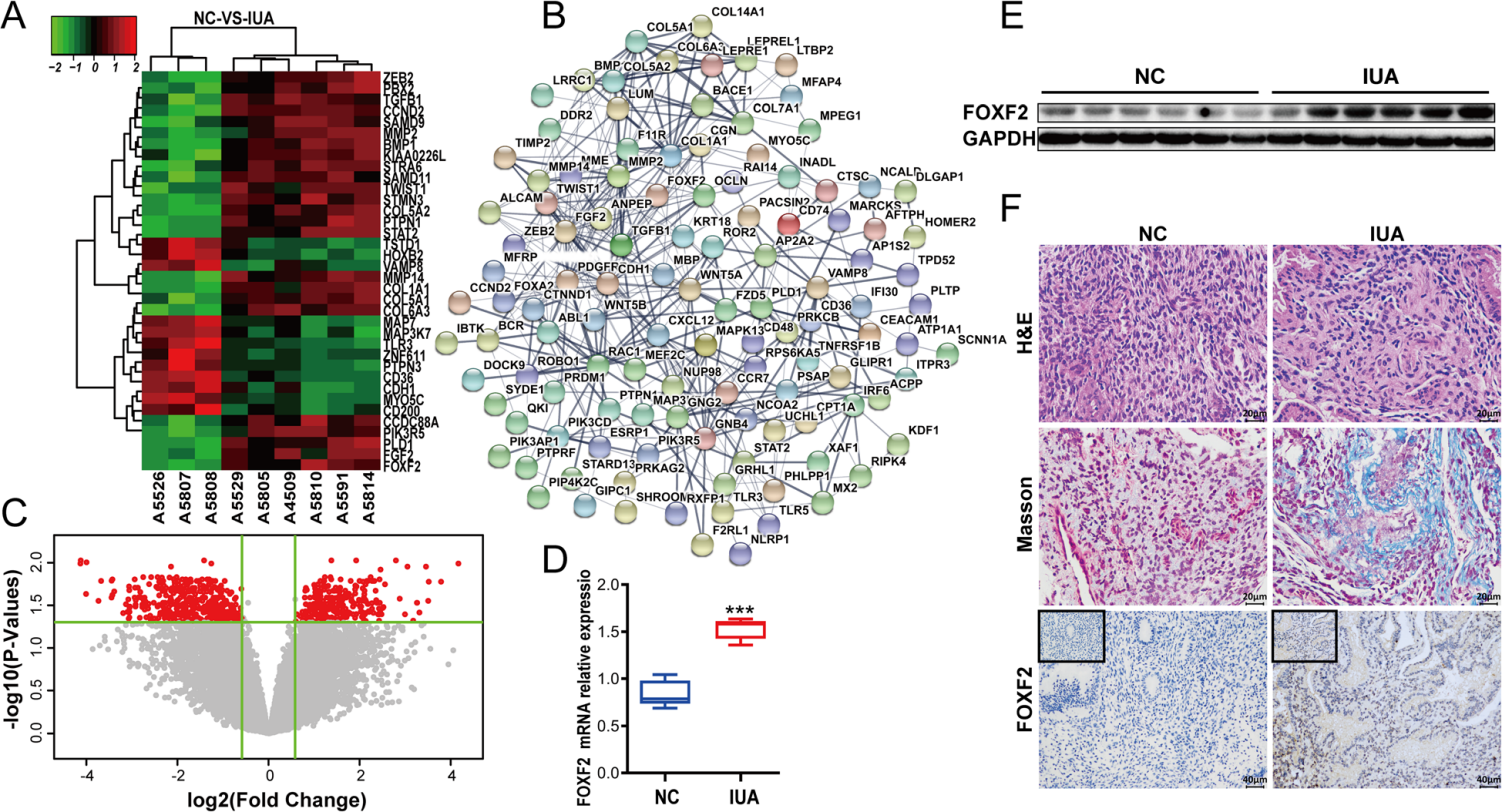
High FOXF2 expression in IUA endometrial tissues. **a** Heat map showing the differentially expressed genes in three samples of normal endometrium and six samples of endometrial adhesions. **b** Protein-protein interaction network constructed with the differentially expressed genes. **c** Volcano plot of differentially expressed genes. **d** FOXF2 mRNA expression in clinical samples measured by qRT-PCR. The measurement data are presented as the means ± SEM, *n* = 6; ***P < 0.001, one-way ANOVA. **e** FOXF2 protein expression in clinical samples. **f** Cell morphology, aggregation of interstitial collagen ,fibres and FOXF2 protein expression in clinical samples

Microarray sequencing analysis was performed to measure the FOXF2 expression levels in two groups of samples, a normal endometrium group and an IUA group. These groups included endometria from three normal subjects and six IUA patients, respectively. FOXF2, TGF-β1 and collagen were more highly expressed in the IUA group than in the normal endometrium group (Fig. 1a). Based on the criteria of *P* < 0.05 and fold change ≥ 1.5, 480 DEGs, including 214 upregulated genes and 266 downregulated genes (Fig. 1c), were identified. The protein relationships among the DEGs were forecasted using STRING database [42]. A PPI network included 194 nodes and 405 edges (Fig. 1b). We further verified the expression of FOXF2 in 12 clinical samples using quantitative reverse transcription-polymerase chain reaction (qRT-PCR), WB and IHC. The results demonstrated that FOXF2 mRNA and protein were expressed at significantly higher levels in the IUA group than in the normal control group (Fig. 1d–f). Masson staining was used to show fibres and inflammatory cells in tissues. The results display collagen fibres in blue and muscle fibres in red, and increased interstitial fibrous tissues and collagen fibres were seen in the endometrium of the IUA group (Fig. 1f).

### Culture and identification of primary HESCs

**Fig. 2:**
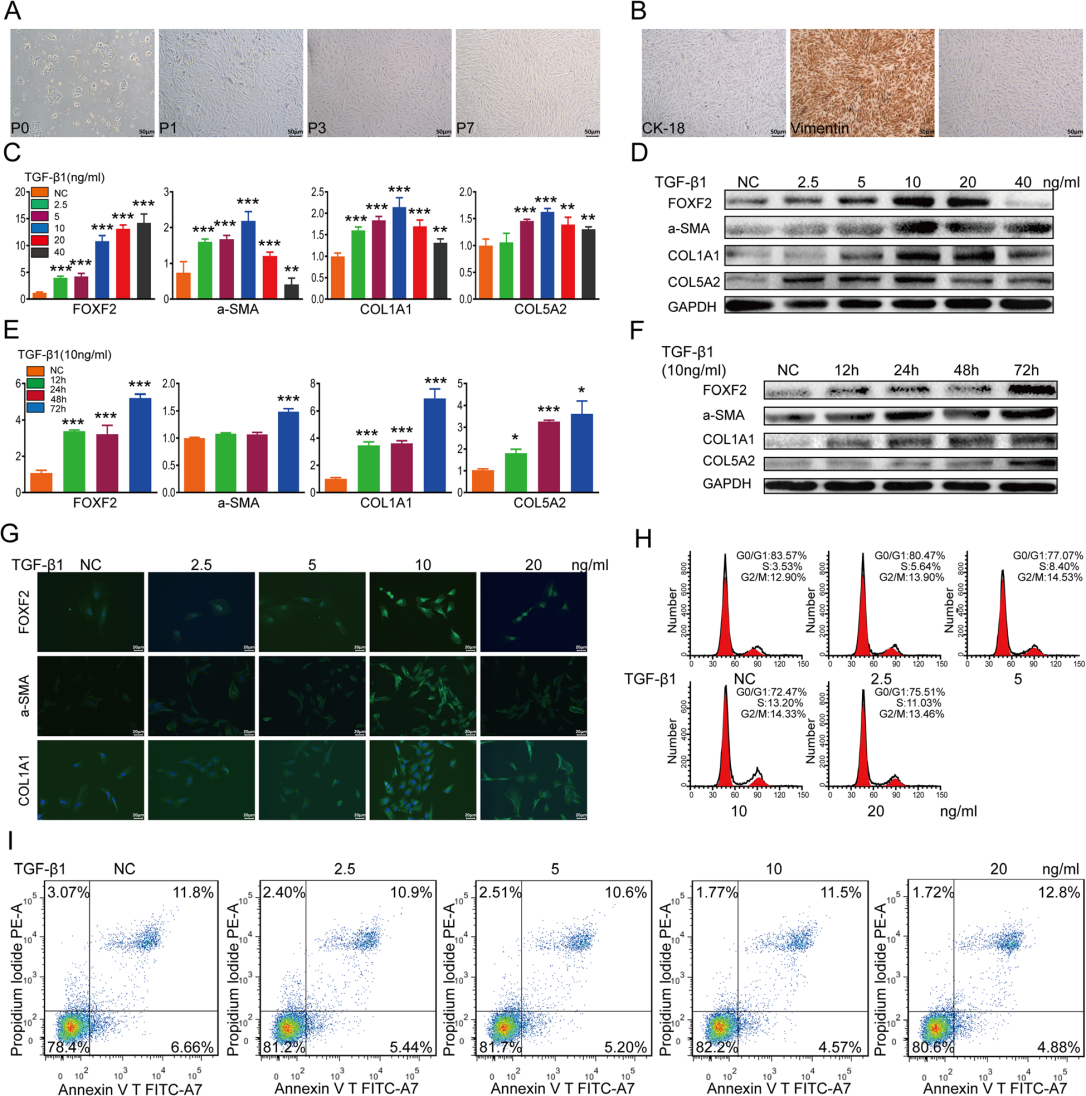
Construction of an IUA cell model using TGF-β1-induced primary HESCs, showing high FOXF2 expression in this model. **a** Morphologies of different generations of primary HESCs. **b** ICC showing the expression levels of VIM and CK-18 in the cytoplasm of primary HESCs. **c** Relative expression of mRNAs in primary HESCs treated with various concentrations of TGF-β1. The measurement data are presented as the means ± SEM, *n* = 3; **P < 0.01, ***P < 0.001, one-way ANOVA. **d** Protein expression in primary HESCs treated with various concentrations of TGF-β1. **e** Relative expression of mRNAs in primary HESCs treated with 10 ng/ml TGF-β1 for different times. The measurement data are presented as the means ± SEM, *n* = 3; *P < 0.05, ***P < 0.001, one-way ANOVA. **f** Protein expression in primary HESCs treated with 10 ng/ml TGF-β1 for various times. **g** IF showing the protein expression in primary HESCs treated with various concentrations of TGF-β1. **h** Flow cytometry analysis showing cell cycle changes after primary HESCs were treated with different concentrations of TGF-β1. **i** Flow cytometry analysis showing that apoptosis was altered in primary HESCs treated with various concentrations of TGF-β1

The morphology of primary HESCs was observed using an inverted microscope. After 24 h, the majority of active primary HESCs became adherent and showed a short fusiform-like fibrous shape. After three generations, the morphology of the HESCs was consistent, and the cells showed fibrous cell-like adherent growth (Fig. 2a). The seventh-generation HESCs maintained a good growth state. Next, we examined the expression of keratin (CK-18) and vimentin (VIM) in the extracted cells to identify primary HESCs. Since CK-18 is mainly present in endometrial epithelial cells (EECs) and VIM is mainly present in endometrial stromal cells (ESCs), we could identify the extracted cells according to their differential expression of CK-18 and VIM. For HESCs, the ICC results showed the presence of VIM in the cytoplasm. Moreover, the purity of the extracted and purified primary HESCs was greater than 95% (Fig. 2b), consistent with results from previous studies [42].

### TGF-β1 induces primary HESC fibrosis (IUA cell model)

TGF-β1 is widely recognized as the key profibrotic factor of fibrosis [43]. Therefore, we used TGF-β1 to induce the transition of primary HESCs into MFs to develop an IUA cell model. First, primary HESCs were treated with various concentrations of TGF-β1 at various times. The qRT-PCR and WB results showed that TGF-β1 induced the conversion of primary HESCs into MFs in a concentration- and time-dependent manner, with increasing the expression of α-SMA, COL1A1, COL5A2 and FOXF2 in HESCs (Fig. 2c–f). Next, we performed IF to further validate elevation of the protein expression levels of α-SMA, COLIA1 and FOXF2 after treatment of the cells with TGF-β1. Notably, α-SMA and COLIA1 were mainly expressed in the cytoplasm, whereas FOXF2 was mainly expressed in the nucleus (Fig. 2g). Flow cytometry analysis was then performed to determine the effects of TGF-β1 on cell proliferation. We found that, when the cells were treated with TGF-β1, the proportion of cells in the G0/G1 phase gradually decreased, while the proportion of cells in the S phase gradually increased. The proportion of early apoptotic cells first gradually decreased and then increased. Taken together, the data demonstrate that TGF-β1 induces the formation of ECM by primary HESCs and promotes cell proliferation. Moreover, treatment of primary HESCs with 10 ng/ml TGF-β1 for 72 h was found to be most effective for developing an IUA cell model (the primer sequences used in these experiments are provided in Supplementary Table 1).

### FOXF2 regulates multiple downstream target genes, including collagen, VIM and cyclin D2/DK4

**Fig. 3:**
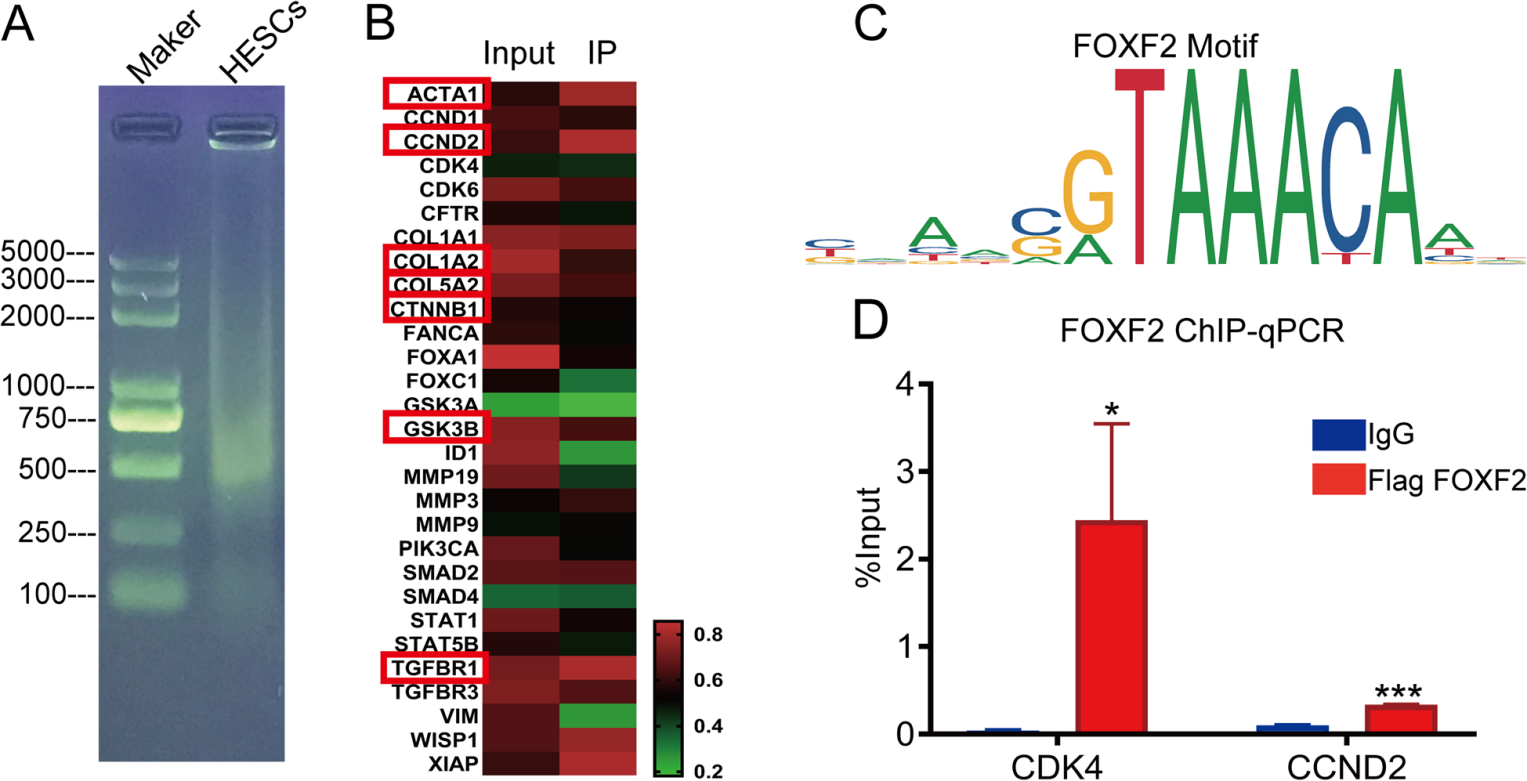
Identification of FOXF2-regulated downstream target genes by ChIP-seq and ChIP-PCR. **a** Agarose gel electrophoretogram showing the distribution of chromosomal DNA fragmentation after ultrasound. **b** Heat map of differentially expressed genes in the ChIP-seq IP and input groups. **c** The FOXF2 motif. **d** ChIP-PCR showing the expression levels of CDK4 and CCND2 in the FOXF2 IP and IgG groups. The measurement data are expressed as the means ± SEM, *n* = 3; *P < 0.05, ***P < 0.001, one-way ANOVA

To clarify how FOXF2 functions in IUA fibrosis, downstream target genes for FOXF2 were screened by chromatin immunoprecipitation combined with whole-genome high-throughput sequencing (ChIP-seq). The heat map results showed that FOXF2 binds to the collagen, ACTA1, VIM, CTNNB1, GSK3B, TGFBR1 and cyclin D2/CDK4 genes and to multiple other genes (Fig. 3b). The ChIP-PCR results indicated that the expression of cyclin D2/CDK4 was significantly higher in the IP group than in the IgG group (Fig. 3d). Indeed, the above genes are closely associated with ECM aggregation or with the cell cycle and the cell’s proliferative status; thus, the results further confirm that changes in FOXF2 levels promote correlated changes in downstream target gene expression and ultimately lead to changes in ECM and cell proliferation. The results indicate that FOXF2 is a potential target for regulating fibrosis by the Wnt/β-catenin pathway (the primer sequences used in these experiments are provided in Supplementary Table 1).

### si-FOXF2 inhibits TGF-β1-mediated primary HESC fibrosis

**Fig. 4:**
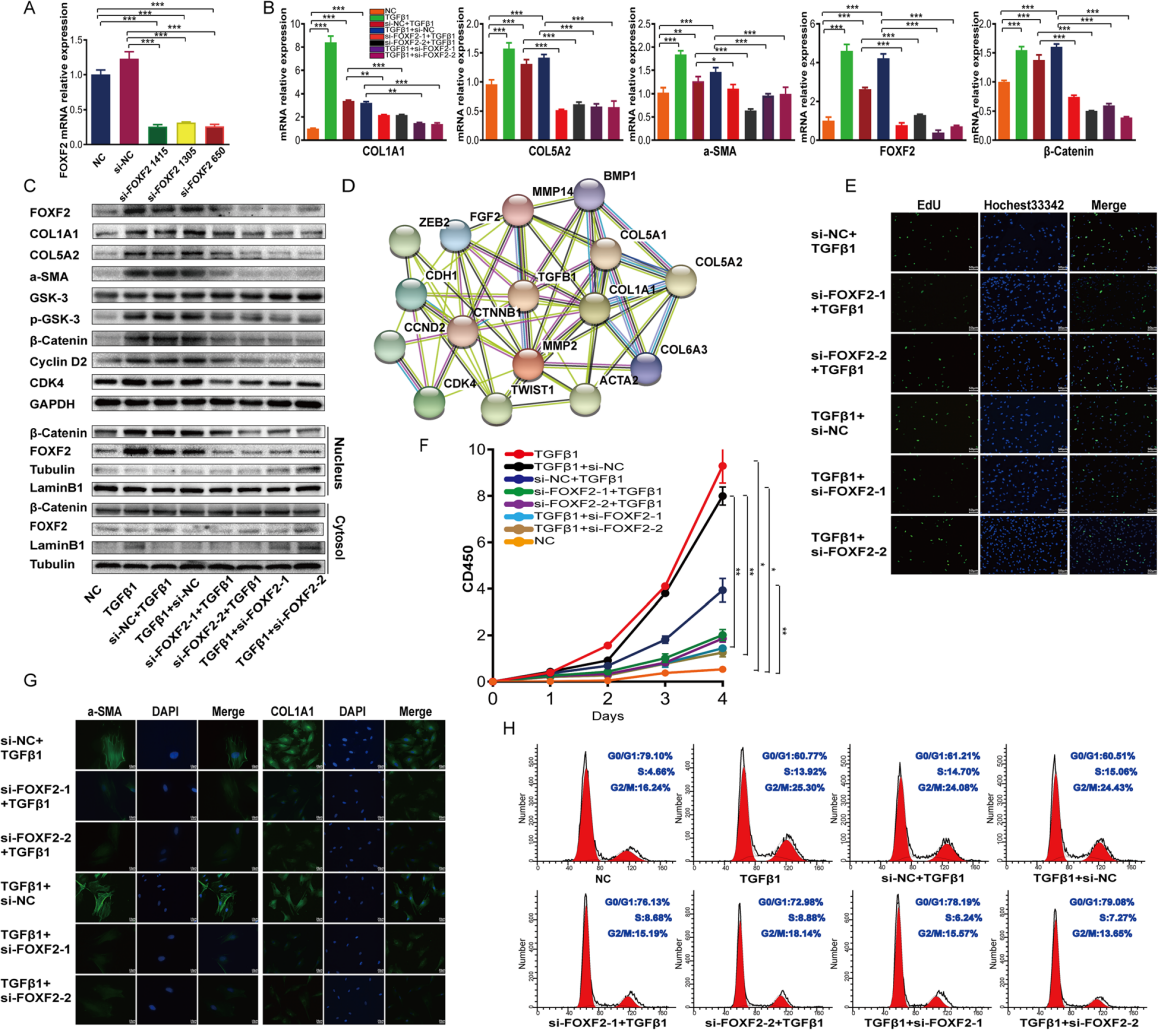
Downregulation of FOXF2 inhibits collagen aggregation, cell proliferation and β-catenin expression in TGF-β1-treated HESCs ex vivo. **a** Relative FOXF2 mRNA expression after si-FOXF2 transfection of primary HESCs. Measurement data are presented as the means ± SEM, *n* = 3; ***P < 0.001, one-way ANOVA. **b** Relative expression of mRNAs in primary HESCs transfected with si-FOXF2 before and after TGF-β1 treatment. Data are expressed as the means ± SEM, *n* = 3; *P < 0.05, **P < 0.01, ***P < 0.001, one-way ANOVA. **c** Protein expression in primary HESCs transfected with si-FOXF2 before and after TGF-β1 treatment. **d** The protein relationship between TGF-β1 and β-catenin was forecasted using STRING tools. **e** EdU assay showing changes in the proliferation of primary HESCs transfected with si-FOXF2 before and after TGF-β1 treatment. **f** CCK8 assay showing the proliferation rate of primary HESCs transfected with si-FOXF2 before and after TGF-β1 treatment. Measurement data are expressed as the means ± SEM, *n* = 3; *P < 0.05, **P < 0.01, one-way ANOVA. **g** IF showing the protein expression levels of α-SMA and COLIA1 in primary HESCs transfected with si-FOXF2 before and after TGF-β1 treatment. **h** Flow cytometry analysis showing changes in the cell cycle of primary HESCs transfected with si-FOXF2 before and after TGF-β1 treatment

To further identify the role of FOXF2 in TGF-β1-mediated primary HESC fibrosis, we used short interfering RNA (siRNA) to downregulate the expression of FOXF2. The qRT-PCR results showed that si-FOXF2 1415 and 650 achieved strong silencing of expression of the respective mRNAs (74% and 73%, respectively). Accordingly, si-FOXF2 1415 and 650 were used in a follow-up intervention experiment in which they were renamed si-FOXF2-1 and si-FOXF2-2, respectively (Fig. 4a). The ChIP-seq and ChIP-PCR results reported above suggest that FOXF2 directly regulates the expression of the collagen, VIM and cyclin D2/CDK4 genes. Based on that, we speculated that si-FOXF2 might inhibit ECM formation and cell proliferation during fibrosis. In this study, primary HESCs transfected with si-FOXF2 before TGF-β1 treatment are referred to as the IUA prevention group, while primary HESCs transfected with si-FOXF2 after TGF-β1 treatment are referred to as the IUA treatment group (the sequences of the primers used in these experiments are provided in Supplementary Table 1, and grouping schemes are provided in Supplementary Table 3).

Using qRT-PCR and WB, we found that the mRNA and/or protein expression levels of FOXF2, α-SMA, COLIA1, COL5A2, p-GSK3, β-catenin and cyclin D2/CDK4 increased significantly in the TGF-β1-induced IUA cell model. We then evaluated the effects of si-FOXF2 in this model. The mRNA and/or protein expression of FOXF2 decreased significantly in the si-FOXF2 IUA prevention group, confirming effective FOXF2 silencing. Marked decreases in the mRNA and/or protein expression of α-SMA, COLIA1, COL5A2, p-GSK3, β-catenin and cyclin D2/CDK4 were observed in the si-FOXF2 IUA prevention group compared to the si-NC prevention group. In contrast, the protein expression of GSK-3, which functions to degrade β-catenin, was increased. The results obtained in the treatment group were consistent with those obtained in the prevention group (Fig. 4b, c). Considering that β-catenin translocates from the cytoplasm to the nucleus and the nature of FOXF2 as a transcription factor whose effects are primarily in the nucleus, we further evaluated the expression of these proteins by WB after separating the nuclear and cytoplasmic fractions. As expected, we found that β-catenin and FOXF2 protein levels in the cytoplasm showed little change in either group. On the other hand, the levels of FOXF2 and β-catenin in the nucleus increased markedly in the IUA cell model and decreased in the si-FOXF2 prevention group. Similar results were obtained in the treatment group (Fig. 4c).

Next, we verified the protein expression of α-SMA and COLIA1 by IF and obtained results consistent with the data described above (Fig. 4d). Furthermore, CCK8 assays and flow cytometry demonstrated that TGF-β1 promoted the proliferation of primary HESCs and that the proliferative effect was counteracted by si-FOXF2 (Fig. 4e, h). An EdU assay was then performed. The results verified the marked decrease in the proportion of cells in S phase (appearing as green fluorescence) in the si-FOXF2 group (Fig. 4e), further supporting the conclusion that si-FOXF2 represses the increased proliferation of primary HESCs caused by TGF-β1.

Crosstalk between the TGF-β1 and Wnt/β-catenin signalling pathways in IUA has remained elusive and poorly demonstrated until now. In this study, the protein relationships among TGF-β1 and the Wnt/β-catenin pathway were explored using STRING tools. The PPI network indicated that the TGF-β1 and β-catenin proteins affect each other (Fig. 4d). These findings support the idea that si-FOXF2 inhibits TGF-β1 activation and suppresses the interaction between TGF-β1 and β-catenin, which may explain the downregulation of β-catenin protein observed in the si-FOXF2 group. We hypothesized that some upstream factors may regulate FOXF2 and β-catenin.

### lncRNA SNHG5 directly regulates FOXF2 expression

**Fig. 5:**
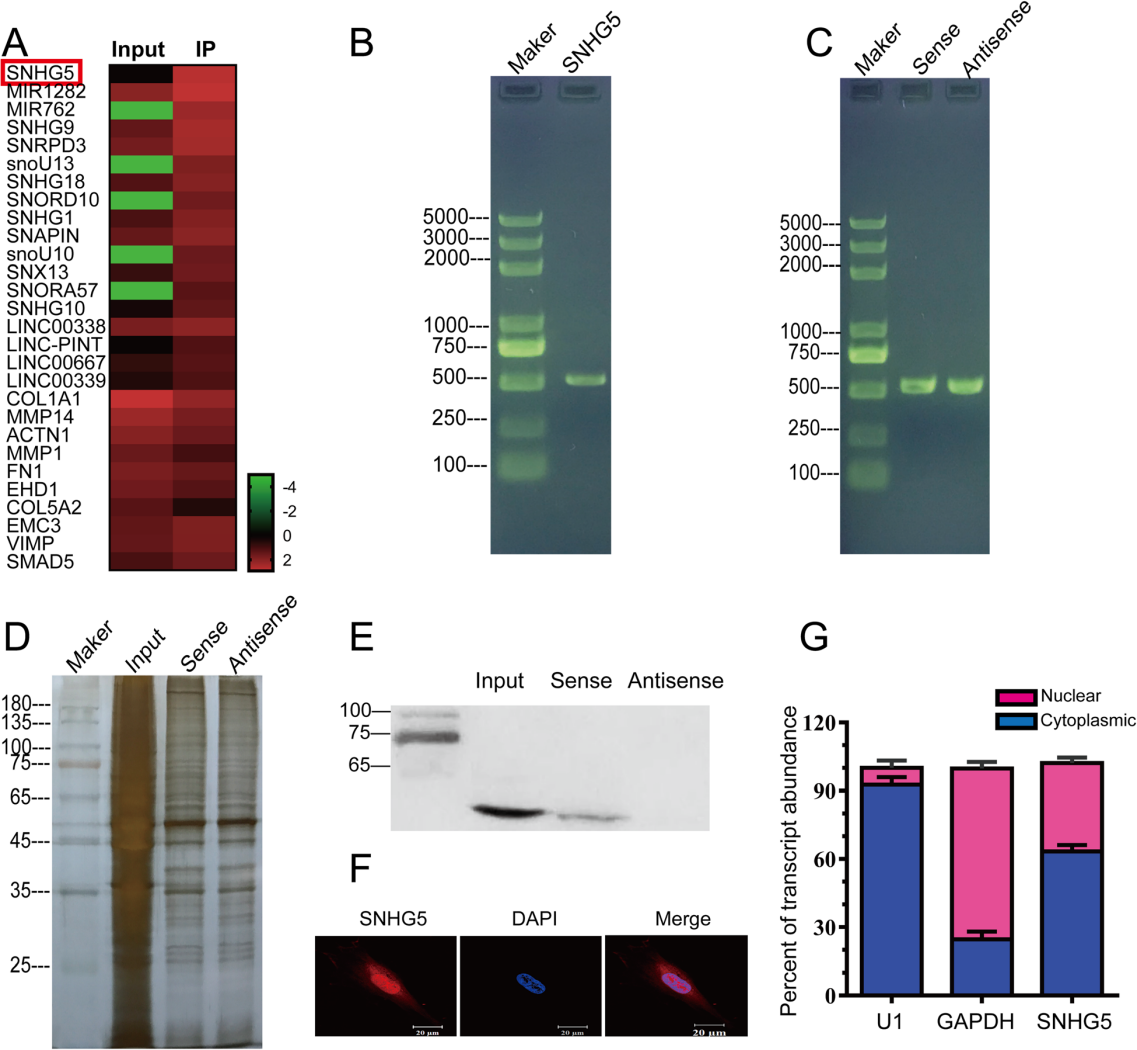
Binding between FOXF2 and lncRNA SNHG5. **a** Heat map of differentially expressed genes in the RIP-seq IP and input groups. **b** Agarose gel electrophoretogram showing the distribution of the amplified SNHG5 fragment. **c** Agarose gel electrophoretogram showing the distribution of the SNHG5 sense and antisense strands. **d** Silver staining after RNA pulldown assay. **e** FOXF2 protein expression after RNA pulldown. **f** Laser scanning confocal microscopy (LSCM) images showing the cellular localization of SNHG5. **g** SNHG5 mRNA expression in the cytoplasm and nucleus. Measurement data are expressed as the means ± standard deviation (SD), *n* = 3

In recent years, studies focused on lncRNAs associated with fibrosis have emerged both in China and abroad. Given the crucial role of the FOXF2 transcription factor in embryonic development, more attention should be paid to identifying alternative therapeutic approaches to inhibit fibrosis than to developing approaches that directly target FOXF2 in vivo. To investigate whether factors upstream of FOXF2 may affect IUA fibrosis, we performed RNA immunoprecipitation and sequencing (RIP-seq) of the FOXF2 protein in primary HESCs. The resulting heat map shows that lncRNA SNHG5 expression was higher in the IP group than in the input group (Fig. 5a). The RNA pulldown, silver staining and WB results confirmed the binding of lncRNA SNHG5 to FOXF2 (Fig. 5b–e). FISH showed that lncRNA SNHG5 was expressed in both the cytoplasm and the nucleus, although its expression was relatively higher in the nucleus (Fig. 5f). That result was further confirmed by qRT-PCR (Fig. 5g; primer sequences are provided in Supplementary Table 1). As mentioned above, the IF results showed high FOXF2 expression in the nucleus (Fig. 2g), in keeping with the nature of FOXF2 as a transcription factor. The finding that both lncRNA SNHG5 and FOXF2 are located in the nucleus provides additional evidence for the occurrence of an interaction between them.

### Inhibition of FOXF2 and regulation of Wnt/β-catenin signalling proteins by si-SNHG5

**Fig. 6:**
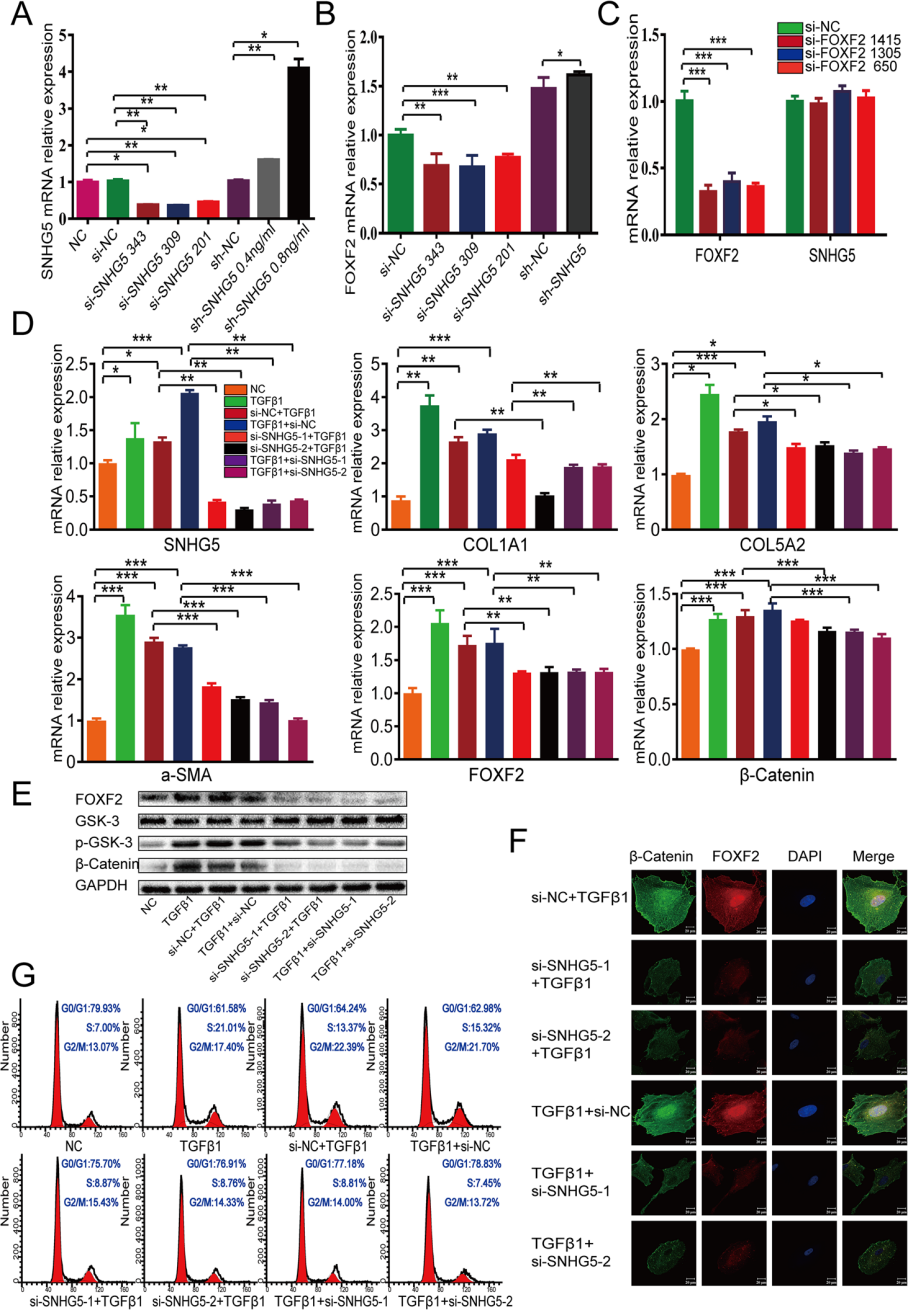
Downregulation of SNHG5 inhibits TGF-β1-mediated HESC fibrosis and the Wnt/β-catenin signalling pathway. **a** Relative expression of SNHG5 mRNA after si-SNHG5 transfection into primary HESCs. Measurement data are expressed as the means ± SEM, *n* = 3; *P < 0.05, **P < 0.01, one-way ANOVA. **b** Relative FOXF2 mRNA expression after si-SNHG5 and sh-SNHG5 transfection into primary HESCs. Measurement data are expressed as the means ± SEM, *n* = 3; *P < 0.05, **P < 0.01, ***P < 0.001, one-way ANOVA. **c** Relative SNHG5 mRNA expression after si-FOXF2 transfection into primary HESCs. Measurement data are expressed as the means ± SEM, *n* = 3; ***P < 0.001, one-way ANOVA. **d** Relative expression of mRNAs in primary HESCs transfected with si-SNHG5 before and after TGF-β1 treatment. The measurement data are expressed as the means ± SEM, *n* = 3; *P < 0.05, **P < 0.01, ***P < 0.001, one-way ANOVA. **e** Protein expression in primary HESCs transfected with si-SNHG5 before and after TGF-β1 treatment. **f** IF showing the protein expression levels of FOXF2 and β-catenin in primary HESCs transfected with si-SNHG5 before and after TGF-β1 treatment. **g** Flow cytometry analysis showing changes in the cell cycle of primary HESCs transfected with si-SNHG5 before and after TGF-β1 treatment

To gain deeper insight into the relationship between SNHG5 and FOXF2 and its effect on the Wnt/β-catenin signalling pathway, siRNA was used to downregulate the expression of SNHG5 and thereby elucidate the mechanism of action of si-SNHG5 in IUA fibrosis. The qRT-PCR results suggested that si-SNHG5 343 and 309 had high silencing mRNA effects (62% and 64%, respectively); therefore, these siRNAs (renamed si-SNHG5-1 and si-SNHG5-2, respectively) were selected for the follow-up intervention experiment. Next, to observe the effects of SNHG5 overexpression on FOXF2, SNHG5 was upregulated in HESCs using SNGH5 expression plasmids applied at concentrations of 0.4 and 0.8 ng/ml. The concentration of 0.8 ng/ml proved to be more effective and was used in the subsequent experiments (Fig. 6a). The qRT-PCR results indicated that the mRNA expression of FOXF2 changed commensurately with SNHG5 (Fig. 6b). However, SNHG5 mRNA expression did not change significantly in the si-FOXF2 groups (Fig. 6c), confirming that SNHG5 regulates FOXF2 positively and unidirectionally. In this study, primary HESCs transfected with si-SNHG5 before TGF-β1 treatment are referred to as the IUA prevention group, and primary HESCs transfected with si-SNHG5 after TGF-β1 treatment are referred to as the IUA treatment group (the sequences of the primers used in these experiments are provided in Supplementary Table 1, and the grouping schemes are described in Supplementary Table 4).

By qRT-PCR and WB, we found that the mRNA and/or protein expression levels of SNHG5, FOXF2, α-SMA, COLIA1, COL5A2, p-GSK3 and β-catenin increased dramatically in the TGF-β1-induced IUA cell model and decreased in the si-SNHG5 prevention group. The effects observed in the IUA treatment group were similar to those observed in the IUA prevention group (Fig. 6d, e). Notably, the expression of GSK-3 in the si-SNHG5 groups was increased, which was in contrast to the decreased expression of β-catenin (Fig. 6e). The IF results showed that β-catenin and FOXF2 protein in the nucleus appeared as hollow vesicles, and a marked decrease in the nuclear aggregation of these proteins was observed in the si-SNHG5 groups (Fig. 6f). Lastly, the flow cytometry results demonstrated that TGF-β1 promoted the proliferation of primary HESCs, whereas the proliferative effect of TGF-β1 was almost completely abrogated by si-SNHG5 (Fig. 6g). Collectively, our data suggest that si-SNHG5, which suppresses TGF-β1 and β-catenin activation, serves as an endogenous mediator of the Wnt/β-catenin pathway and thereby regulates the TGF-β1-induced primary activation of HESCs in fibrosis.

## Discussion

IUAs are manifestations of endometrial fibrosis caused by factors such as trauma or infection. The main pathological changes that occur in fibrosis are inflammation, the production of ECM and activation of MFs in the EMT process [17]. Frustratingly, in clinical practice, IUAs are unpredictable and thus difficult to prevent. When symptoms and signs occur, such as decreased menstrual flow and infertility, IUAs are often already present; on the other hand, the therapeutic outcomes of treatment for IUAs are not currently encouraging.

TGF-β1 is a cytokine that is well-recognized as a key driver in the development of fibrotic diseases [43]. When kidney tissue experiences sustained injury, epithelial cells continuously secrete a large amount of TGF-β1, inducing epigenetic changes in FBs and the transformation of FBs into tumour-like MFs [44]. The establishment of an IUA animal model in our previous studies demonstrated that the classical fibrogenic factor TGF-β1 participates in the pathogenesis of IUAs via the TGF-β/Smad signalling pathway [39, 40]. Our increased understanding of how TGF-β and its interacting factors regulate fibrosis allowed us to identify a number of latent antifibrotic targets that might be used to avoid or retard the development of fibrotic disease [10]. Using genome-wide microarray analysis, we found that TGF-β1 and collagen were more highly expressed in IUA clinical samples than in clinical samples of normal endometrium. The studies and results described above motivated us to use TGF-β1 to induce the transition of primary HESCs into MFs to develop an ex vivo IUA cell model and to explore the molecular mechanism underlying the inhibition of TGF-β1-mediated endometrial fibrosis.

Our study confirmed that ECM aggregation increased in the IUA cell model and that primary HESCs proliferated abnormally and excessively ex vivo. In this regard, TGF-β1 may be considered a target that could be directly inhibited to reduce ECM formation and further decrease fibrosis. However, TGF-β1 regulates many biological reactions other than organ fibrosis; these reactions involve cell proliferation, differentiation, autophagy, apoptosis and the immune response [45]. Furthermore, TGF-β1 is speculated to play an important dual role in epithelial tissue repair that may not be substitutable [14]. Therefore, suppression of TGF-β1 function could aggravate autoimmune diseases through the lack of a process connected with the inhibition of TGF-β in regulatory T cells. At the same time, progress in translating findings from basic studies to clinical application has been very slow [10]. In truth, apprehension about the potential adverse outcomes of targeting TGF-β1 has spurred many investigators to attempt to discover the pathways that underlie TGF-β1-mediated fibrosis with the aim of developing alternative therapeutic strategies to inhibit tissue fibrosis. We took a similar approach in this study. Compared with the normal endometrium group, we found that FOXF2, TGF-β1 and collagen were more highly expressed in whole-genome microarray data from the IUA group. TGF-β1 stimulated the increased expression of FOXF2 in HESCs ex vivo, indicating that FOXF2 is implicated in the formation of IUAs and could be a potential target.

To clarify how FOXF2 functions in IUA fibrosis, downstream target genes for FOXF2 were screened by ChIP-seq. The results showed that FOXF2 binds to collagen, VIM, cyclin D2/CDK4 and multiple other genes. We downregulated the expression of FOXF2 and further elucidated the mechanism of si-FOXF2 in IUA fibrosis. Marked decreases in the mRNA and/or protein expression of α-SMA, COLIA1, COL5A2, p-GSK3, β-catenin and cyclin D2/CDK4 were observed in the si-FOXF2 IUA prevention or treatment groups. Moreover, the results demonstrated that si-FOXF2 could counteract the effects of TGF-β1-promoted primary HESC proliferation and ECM aggregation. The above results indicate that downregulation of FOXF2 inhibits TGF-β1-mediated primary HESC fibrosis.

Some studies have shown that the canonical Wnt/β-catenin signalling is required for TGF-β1-mediated fibrosis, a finding that highlights the key interaction between the Wnt/β-catenin and TGF-β signalling pathways in the pathogenesis of fibrotic diseases [18, 46]. However, crosstalk between the TGF-β1 and Wnt/β-catenin signalling pathways in IUA has remained elusive and poorly demonstrated until now. In our study, increased expression of p-GSK3, β-catenin and cyclin D2/CDK4; accumulation of ECM; and abnormal cell proliferation were observed in the ex vivo IUA cell model. These findings confirmed that the Wnt/β-catenin signalling pathway is necessary for TGF-β1-mediated HESC fibrosis and that TGF-β1 activates the canonical Wnt pathway. It was reported that si-FOXF2 stimulated WNT1 and β-catenin and further upregulated the Wnt target genes cyclin D and c-myc in a gastric cancer cell line [47]. We also found that the protein expression levels of p-GSK3 and β-catenin, as well as the nuclear aggregation of β-catenin, decreased after FOXF2 gene silencing in the ex vivo IUA cell model. We speculated that downregulation of FOXF2 antagonizes the effect of TGF-β1 and further strongly reduces activation of the canonical Wnt/β-catenin signalling pathway. Despite our recognition of the profibrotic effect of FOXF2, absolute inhibition of FOXF2 may be risky. A study reported that FOXF2^−/−^ mice die at birth due to a lack of exons encoding the DNA-binding domain [23]. Given the critical role of the FOXF2 transcription factor in embryonic development, we should emphasize the investigation of alternative therapeutic approaches to inhibit IUA that are not based on direct targeting of FOXF2 in vivo. At the same time, we hypothesize that additional factors may regulate FOXF2 in the nucleus and β-catenin in the cytoplasm.

An increasing number of lncRNAs have been implicated in the initiation and development of fibrotic diseases, and the related mechanisms have been confirmed and elucidated by researchers. However, no reports concerning lncRNAs associated with IUAs have been available until now. In the present study, we used RIP-seq to screen out lncRNA SNHG5, which binds tightly to the FOXF2 protein, and further demonstrated by RNA pulldown and WB that FOXF2 and lncRNA SNHG5 bind to each other. This study also showed that lncRNA SNHG5 was highly expressed in the IUA cell model and that it positively regulated FOXF2. Both lncRNA SNHG5 and FOXF2 are closely associated with IUAs. In addition, our findings showed that both lncRNA SNHG5 and FOXF2 are located in the nucleus, providing additional evidence of the interaction between them. A study showed that knockdown of SNHG5 remarkably inhibited GSK3β and the Wnt/β-catenin pathway in hepatocellular carcinoma (HCC) or brain glioma cell lines [38, 48]. Similar results were obtained in our study, as knockdown of lncRNA SNHG5 resulted in decreased protein expression of FOXF2, p-GSK3 and β-catenin; intranuclear aggregation of β-catenin; ECM aggregation; and abnormal cell proliferation in the ex vivo IUA cell model. Based on these results, we concluded that regulation of the Wnt/β-catenin signalling pathway and ECM formation by si-SNHG5-FOXF2 effectively inhibited the profibrotic effect of TGF-β1 on HESCs. This study focused on the regulation of Wnt/β-catenin pathway expression by SNHG5 and FOXF2 in TGF-β1-induced fibrosis at the cellular level and the corresponding transcriptomic mechanism. Thus, the results provide a theoretical basis for the biological mechanism through which IUAs occur.

## Conclusions

Targeted treatment for endometrial fibrosis during the formation of IUA is needed due to the discouraging therapeutic outcomes of this disease at present. Our study offers several convincing lines of evidence that lncRNAs SNHG5 and FOXF2 play an important role in regulating TGF-β1-mediated primary HESC fibrosis, mainly through activating canonical Wnt/β-catenin signalling. Consequently, lncRNAs SNHG5 and FOXF2 represent potential targets for the prevention and treatment of endometrial fibrosis in IUAs. Nevertheless, future studies are required to thoroughly understand the regulatory mechanism of si-SNHG5-FOXF2 in IUA and further translate these hopeful preclinical findings into valid therapeutic agents for the treatment of IUA.

## Supplementary Information

**Additional file 1: Table S1.** mRNA primer sequences. **Table S2**. The antibodies used in WB, ICC, IF, and IP. **Table S3**. The treatment of each group of HESCs with si-FOXF2. **Table S4.** The treatment of each group of HESCs with si-SNHG5.

**Additional file 2: Figure S1.** protein expression in clinical samples or HESCs. (A) FOXF2 protein expression in clinical samples. (B-E) Protein expression in primary HESCs treated with various concentrations of TGF-β1. *P < 0.05, ** *P* < 0.01, *** *P* < 0.001 vs normal group.

**Additional file 3: Figure S2.** Protein expression, cell cycle and apoptosis in TGF-β1-induced primary HESCs. (A-D) Protein expression in primary HESCs treated with 10 ng/ml TGF-β1 for various times. (E) Cell cycle changes in primary HESCs treated with different concentrations of TGF-β1. (F) Cell apoptosis changes in primary HESCs treated with various concentrations of TGF-β1.*P < 0.05, ** *P* < 0.01, *** *P* < 0.001 vs normal group.

**Additional file 4: Figure S3.** Protein expression in primary HESCs transfected with si-FOXF2 before and after TGF-β1 treatment. (A) FOXF2; (B) α-SMA; (C) COL1A1; (D) COL5A2; (E) GSK-3; (F) p-GSK-3; (G) β-catenin; (H) Cyclin D2; (I) CDK4. *P < 0.05, ** *P* < 0.01, *** *P* < 0.001 between two groups.

**Additional file 5: Figure S4.** Protein expression, cell proliferation and cell cycle in primary HESCs transfected with si-FOXF2 before and after TGF-β1 treatment. (A) The FOXF2, α-SMA and COL1A1 nuclear protein expression. (B) The FOXF2, α-SMA and COL1A1 cytosolic protein expression. (C) EdU assay showing changes in the proliferation of primary HESCs transfected with si-FOXF2 before and after TGF-β1 treatment. (D) Cell cycle changes in primary HESCs treated with si-FOXF2 before and after TGF-β1 treatment. *P < 0.05, ** *P* < 0.01, *** *P* < 0.001 between two groups.

**Additional file 6: Figure S5.** Protein expression and cell cycle in primary HESCs transfected with si-SNHG5 before and after TGF-β1 treatment. (A) FOXF2, (B) p-GSK-3, (C)GSK-3 and (D) β-catenin protein expression. (E) Cell cycle changes in the proliferation of primary HESCs transfected with si-SNHG5 before and after TGF-β1 treatment. *P < 0.05, ** *P* < 0.01, *** *P* < 0.001 between two groups.

## Data Availability

The data that support the findings of this study are available upon request to the corresponding author.

## Abbreviations

IUAs: Intrauterine adhesions
ECM: Extracellular matrix
TGF-β1: Transforming growth factor-β1
MAPK: Mitogen-activated protein kinase
FBs: Fibroblasts
α-SMA: α-Smooth muscle actin
MFs: Myofibroblasts
HESCs: Human endometrial stromal cells
FOXF2: Transcription factor forkhead box F2
EMT: Epithelial-mesenchymal transition
lncRNA: Long noncoding RNA
snoRNA: Small nucleolar RNA
ChIP-seq: Chromatin immunoprecipitation combined with whole-genome high-throughput sequencing
RIP: RNA immunoprecipitation
PPI: Protein-protein interaction
VIM: Vimentin
ESCs: Endometrial stromal cells
EECs: Endometrial epithelial cells
COL1A1: Collagen IA1
COL5A2: Collagen VA2
CDK: Cyclin-dependent kinase
GSK-3: Glycogen synthase kinase
