# Supplementary material for: si-SNHG5-FOXF2 inhibits TGF-β1-induced fibrosis in human primary endometrial stromal cells by the Wnt/β-catenin signalling pathway
Source: Stem Cell Res Ther. 2020 Nov 11;11:479. doi: 10.1186/s13287-020-01990-3 (PMC7656702; doi:10.1186/s13287-020-01990-3)
Supplement: Supplementary file 1 — Additional file 1: Table S1. mRNA primer sequences. Table S2. The antibodies used in WB, ICC, IF, and IP. Table S3. The treatment of each group of HESCs with si-FOXF2. Table S4. The treatment of each group of HESCs with si-SNHG5. [file 13287_2020_1990_MOESM1_ESM.docx]

Additional file 1

**Supplemental Table1：mRNA primer sequences**

| **Name** | | **Sequence(5’-3’)** |
| --- | --- | --- |
| COL1A1  FOXF2  α-SMA  COL5A2  GAPDH  si-FOXF2 1415  si-FOXF2 1305  si-FOXF2 650  si-NC  si-SNHG5 343  si-SNHG5 309  si-SNHG5 201  β-catenin  SNHG5  SNHG5-T7    U1 | Forward primer:  Reverse primer:  Forward primer:  Reverse primer:  Forward primer:  Reverse primer:  Forward primer:  Reverse primer:  Forward primer:  Reverse primer:  Forward primer:  Reverse primer:  Forward primer:  Reverse primer:  Forward primer:  Reverse primer:  Forward primer:  Reverse primer:  Forward primer:  Reverse primer:  Forward primer:  Reverse primer:  Forward primer:  Reverse primer:  Forward primer:  Reverse primer:  Forward primer:  Reverse primer:  Sense Forward primer:  Antisense Forward primer:  Forward primer:  Reverse primer: | 5'-GAGGGCCAAGACGAAGACATC-3' 5'-CAGATCACGTCATCGCACAAC-3'  5'-TCGCTGGAGCAGAGCTACTT-3'  5'-CCCATTGAAGTTGAGGACGA-3'  5'-GGCTCTGGGCTCTGTAAGG-3'  5'-CTCTTGCTCTGGGCTTCATC-3'  5'-TCTTGCTCCTGTGGATGTTG-3'  5'-TTGATGGTGGTGCTCATTGT-3'  5'-CGGAGTCAACGGATTTGGTCGTAT-3'  5'-AGCCTTCTCCATGGTGGTGAAGAC-3'  5'-GCGUCUGUCAGGAUAUUAATT-3'  5'-UUAAUAUCCUGACAGACGCTT-3'  5'-GCAUCACUCUACUCCAGUGTT-3'  5'-CACUGGAGUAGAGUGAUGCTT-3'  5'-CCAGCGAGUUAUGUUCGATT-3'  5'-UCGAACAUGAACUCGCUGGTT-3'  5'-UUCUUCGAACGUGUCACGUTT-3' 5'-ACGUGACACGUUCGGAGAATT-3'  5'-CUUGACUGUUGUGUGAAAATT-3'  5'-UUUUCACACAACAGUCAAGTT-3'  5'-GCAACGAUUUCUGGCUAGUTT-3'  5'-ACUAGCCAGAAAUCGUUGCTT-3'  5'-CAGUGAAGAUAAUGAAUGUTT-3'  5'-ACAUUCAUUAUCUUCACUGTT-3'  5’-ATGGCTTGGAATGAGACTGC-3’  5’-CTGGCCATATCCACCAGAGT-3’  5'- GGGTGGTAGGAACAATGGCG-3'  5'- CTCGTCCACACTCAGAACGC-3'  5'-GTAATACGACTCACTATAGGGCTTTTACGTCGGCCTTCGC-3'  5'-GTAATACGACTCACTATAGGGAGTGGATTTTCCATTTAATGCTCC-3'  5'-GGGAGATACCATGATCACGAAGGT-3'  5'-CCACAAATTATGCAGTCGAGTTTCCC-3 |

**Supplemental Table2：The antibodies used in WB,ICC,IFand IP**

| **Name** | **Sequence(5’-3’)** |
| --- | --- |
| anti-COLIA1 antibody  anti-α-SMA antibody  anti-COL5A2 antibody  anti-FOXF2 antibody  anti-cytokeratin 18 antibody  anti-vimentin antibody  anti-GAPDH antibody  anti-CDK4 antibody  anti-β-catenin antibody  anti-GSK3 antibody  anti-p-GSK3 antibody  anti-cyclinD2 antibody  anti-Lamin B1 antibody  anti-α/β-Tubulin antibody  secondary antibody | WB: CST#84336,1:1 000;IF: Santa Cruz Biotechnology 28657,1:50  WB: CST #19245,1:1 000;IF: CST #19245,1:200  WB: Abcam ab7046,1:1 000  WB: Abcam ab194427,1:500;IF: Abcam ab194427,1:100;IHC: Abcam ab194427,1:50,IP: Abcam  ab23306 1.2µg/ml;  ICC: Santa Cruz Biotechnology,1:100  ICC: Santa Cruz Biotechnology,1:100  WB: CST,1:3 000  WB: CST #12790,1:1 000  WB: CST #8480,1:1 000;IF: CST #8480,1:100  WB: CST #12456,1:1000  WB: CST #5558,1:1000  WB: CST #3741,1:1000  WB: CST #12586,1:1000  WB: CST #3741,1:1000  WB: House Anti Mouse,CST 1:3000;Goat Anti Rabbit,CST 1:3000;  Mouse Anti Goat, Millipore 1:10 000  IF:(ALexa Fluor™ 488) Goat Anti Rabbit IgG、Goat Anti Mouse IgG、Rabbit Anti Goat IgG, invitrogen,1:200;(ALexa Fluor™ 633) Goat Anti Mouse IgG, Thermo Fisher A-21050,1:200 |

**Supplemental Table3. The treatment of each group HESCs with si-FOXF2**

| **Group** | **Treatment** |
| --- | --- |
| NC：  TGF-β1 treatment：  si-NC preventive treatment：  si-NC therapeutic treatment：  si-FOXF2-1 preventive treatment：  si-FOXF2-2 preventive treatment：  si-FOXF2-1 therapeutic treatment：  si-FOXF2-2 therapeutic treatment： | The HESCs were incubated without TGF-β1.  The HESCs were stimulated with 10ng/ml TGF-β1.  The HESCs were transfected with 100nmol/ml si-NC 12h and then stimulated with 10ng/ml TGF-β1.  The HESCs were stimulated with 10ng/ml TGF-β1 48h ahead of 100nmol /ml si-NC transfected 12h and then stimulated with 10ng/ml TGF-β1.  The HESCs were transfected with 100nmol/ml si-FOXF2-1  12h and then stimulated with 10ng/ml TGF-β1.  The HESCs were transfected with 100nmol/ml si-FOXF2-2  12 h and then stimulated with 10ng/ml TGF-β1.  The HESCs were stimulated with 10ng/ml TGF-β1 48h  ahead of 100nmol /ml si-FOXF2-1transfected 12h and then  were continuously stimulated with 10ng/ml TGF-β1.  The HESCs were stimulated with 10ng/ml TGF-β1 48h  ahead of 100nmol /ml si-FOXF2-2 transfected 12h and then  were continuously stimulated with 10ng/ml TGF-β1. |

**Supplemental Table4. The treatment of each group HESCs with si-SNHG5**

| **Group** | **Treatment** |
| --- | --- |
| NC：  TGF-β1 treatment：  si-NC preventive treatment：  si-NC therapeutic treatment：  si-SNHG5-1 preventive treatment：  si-SNHG5-2 preventive treatment：  si-SNHG5-1 therapeutic treatment：  si-SNHG5-2 therapeutic treatment： | The HESCs were incubated without TGF-β1.  The HESCs were stimulated with 10ng/ml TGF-β1.  The HESCs were transfected with 100nmol/ml si-NC 12h and then stimulated with 10ng/ml TGF-β1.  The HESCs were stimulated with 10ng/ml TGF-β1 48h ahead of 100nmol /ml si-NC transfected 12h and then stimulated with 10ng/ml TGF-β1.  The HESCs were transfected with 100nmol/ml si-SNHG5-1  12h and then stimulated with 10ng/ml TGF-β1.  The HESCs were transfected with 100nmol/ml si-SNHG5-2  12 h and then stimulated with 10ng/ml TGF-β1.  The HESCs were stimulated with 10ng/ml TGF-β1 48h  ahead of 100nmol /ml si-SNHG5-1transfected 12h and then  were continuously stimulated with 10ng/ml TGF-β1.  The HESCs were stimulated with 10ng/ml TGF-β1 48h  ahead of 100nmol /ml si-SNHG5-2 transfected 12h and then  were continuously stimulated with 10ng/ml TGF-β1. |
