# Supplementary figures and images for: si-SNHG5-FOXF2 inhibits TGF-β1-induced fibrosis in human primary endometrial stromal cells by the Wnt/β-catenin signalling pathway
Source: Stem Cell Res Ther. 2020 Nov 11;11:479. doi: 10.1186/s13287-020-01990-3 (PMC7656702; doi:10.1186/s13287-020-01990-3)

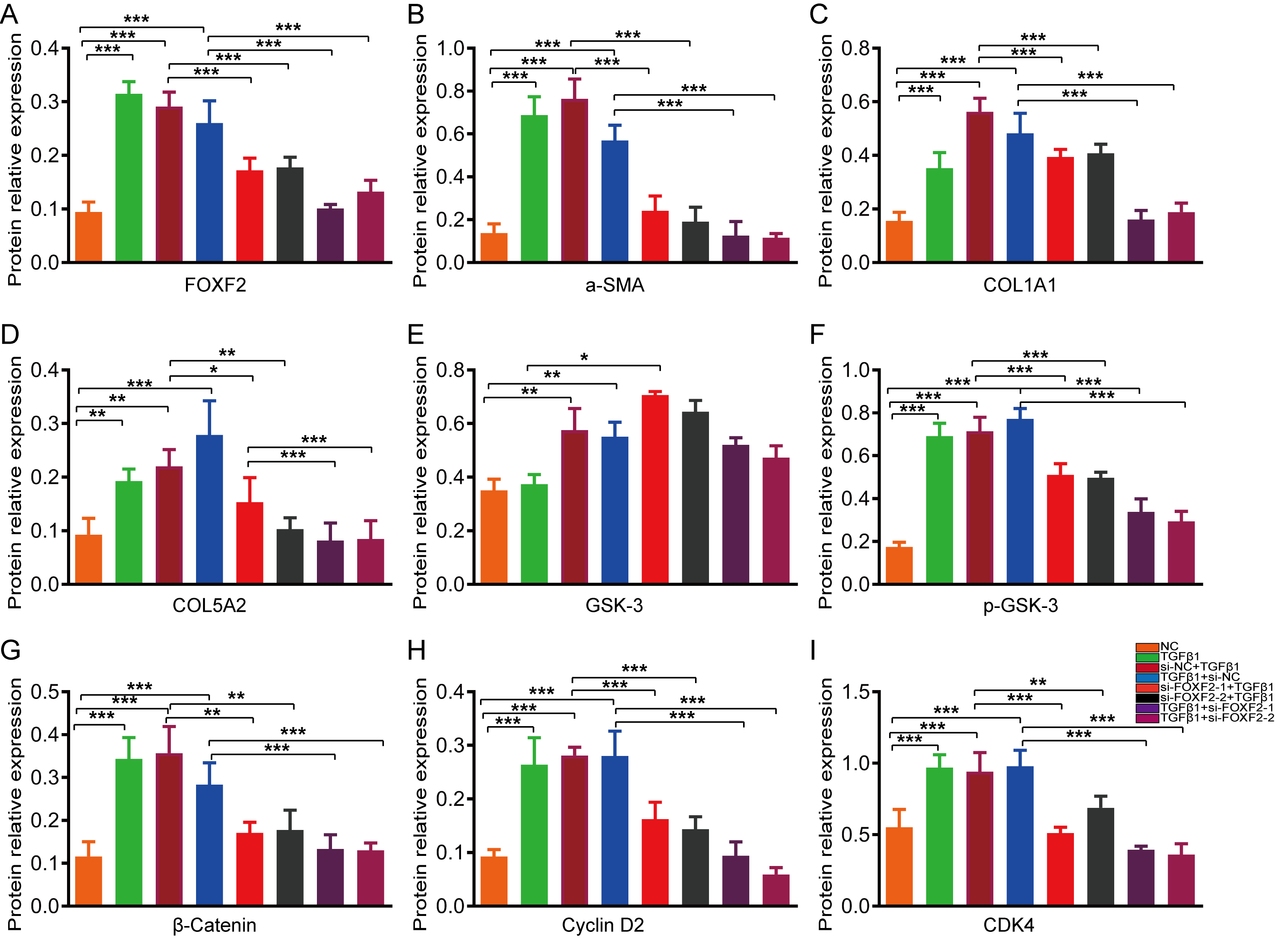

Supplement: Supplementary file 4 — Additional file 4: Figure S3. Protein expression in primary HESCs transfected with si-FOXF2 before and after TGF-β1 treatment. (A) FOXF2; (B) α-SMA; (C) COL1A1; (D) COL5A2; (E) GSK-3; (F) p-GSK-3; (G) β-catenin; (H) Cyclin D2; (I) CDK4. *P < 0.05, ** P < 0.01, *** P < 0.001 between two groups. [file 13287_2020_1990_MOESM4_ESM.tif]

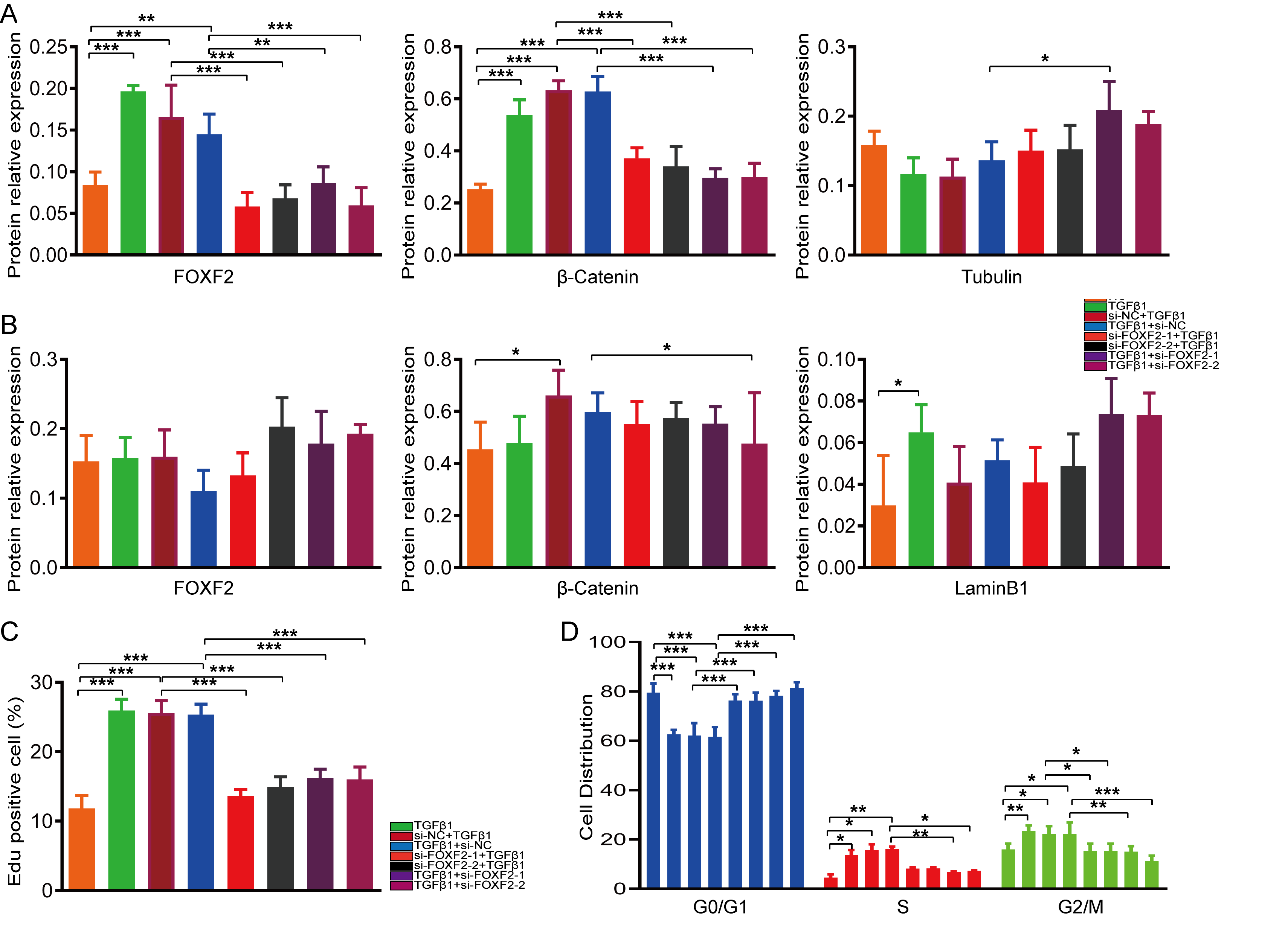

Supplement: Supplementary file 5 — Additional file 5: Figure S4. Protein expression, cell proliferation and cell cycle in primary HESCs transfected with si-FOXF2 before and after TGF-β1 treatment. (A) The FOXF2, α-SMA and COL1A1 nuclear protein expression. (B) The FOXF2, α-SMA and COL1A1 cytosolic protein expression. (C) EdU assay showing changes in the proliferation of primary HESCs transfected with si-FOXF2 before and after TGF-β1 treatment. (D) Cell cycle changes in primary HESCs treated with si-FOXF2 before and after TGF-β1 treatment. *P < 0.05, ** P < 0.01, *** P < 0.001 between two groups. [file 13287_2020_1990_MOESM5_ESM.tif]

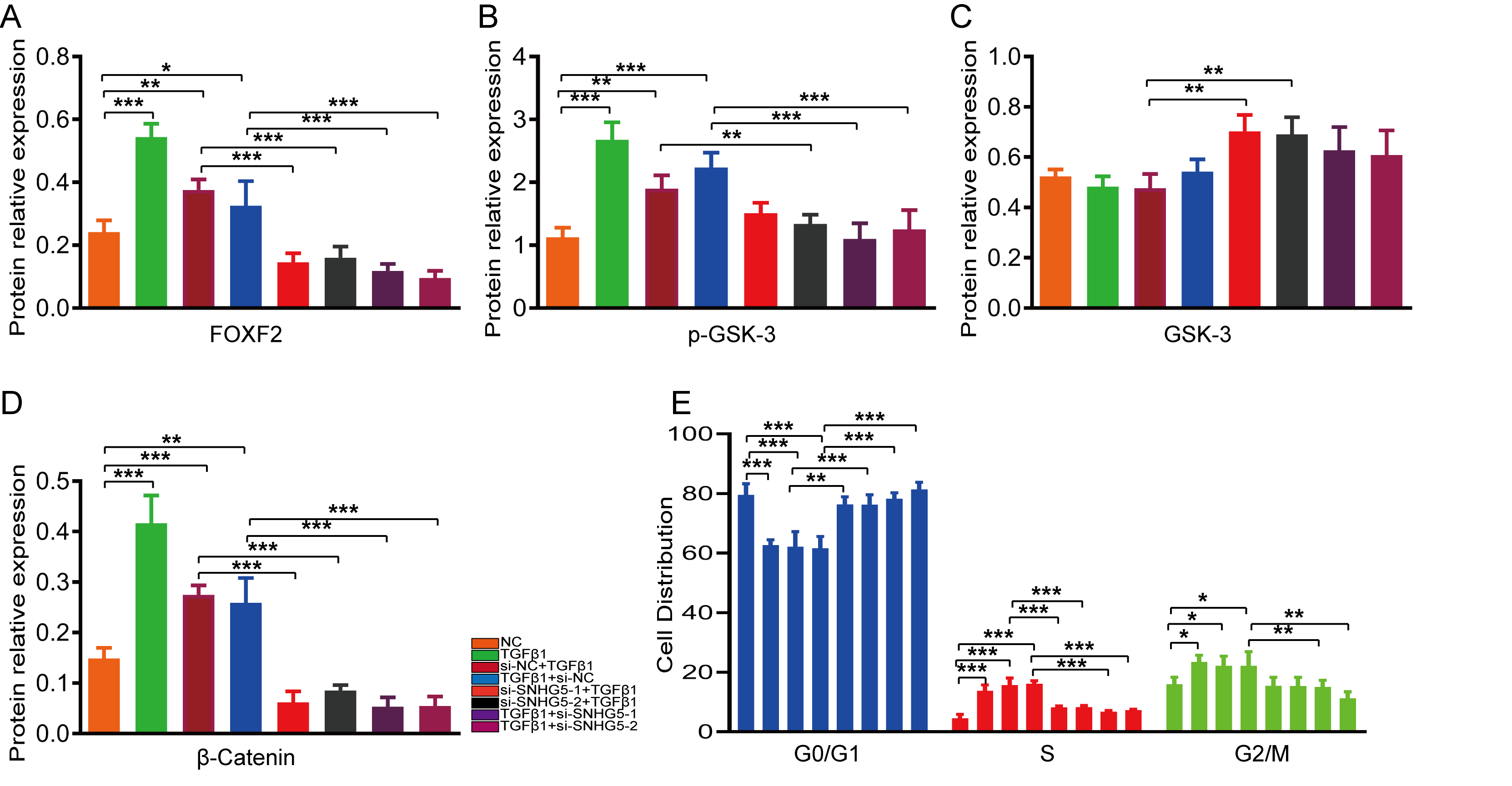

Supplement: Supplementary file 6 — Additional file 6: Figure S5. Protein expression and cell cycle in primary HESCs transfected with si-SNHG5 before and after TGF-β1 treatment. (A) FOXF2, (B) p-GSK-3, (C)GSK-3 and (D) β-catenin protein expression. (E) Cell cycle changes in the proliferation of primary HESCs transfected with si-SNHG5 before and after TGF-β1 treatment. *P < 0.05, ** P < 0.01, *** P < 0.001 between two groups. [file 13287_2020_1990_MOESM6_ESM.tif]
